# Structural Characterization and Interaction with RCA_120_ of a Highly Sulfated Keratan Sulfate from Blue Shark (*Prionace glauca*) Cartilage

**DOI:** 10.3390/md16040128

**Published:** 2018-04-14

**Authors:** Qinying Li, Guoyun Li, Xiaoliang Zhao, Xindi Shan, Chao Cai, Jing Zhao, Fuming Zhang, Robert J. Linhardt, Guangli Yu

**Affiliations:** 1 Key Laboratory of Marine Drugs, Ministry of Education, Shandong Provincial Key Laboratory of Glycoscience and Glycotechnology, School of Medicine and Pharmacy, Ocean University of China, Qingdao 266003, China; liqinying2010@163.com (Q.L.); zhxl819@163.com (X.Z.); shanxindi@gmail.com (X.S.); caic@ouc.edu.cn (C.C.); 2 Laboratory for Marine Drugs and Bioproducts, Qingdao National Laboratory for Marine Science and Technology, Qingdao 266003, China; 3 Department of Chemistry and Chemical Biology, Biomedical Engineering, Biology, Chemical and Biological Engineering, and Center for Biotechnology and Interdisciplinary Studies, Rensselaer Polytechnic Institute, Troy, NY 12180, USA; zhaoj10@rpi.edu (J.Z.); zhangf2@rpi.edu (F.Z.); linhar@rpi.edu (R.J.L.)

**Keywords:** *Prionace glauca* cartilage, keratan sulfate, structural characterization, NMR spectra, HILIC-FTMS, *Ricinus communis* agglutinin I

## Abstract

As an important glycosaminoglycan, keratan sulfate (KS) mainly exists in corneal and cartilage, possessing various biological activities. In this study, we purified KS from blue shark (*Prionace glauca*) cartilage and prepared KS oligosaccharides (KSO) through keratanase II-catalyzed hydrolysis. The structures of KS and KSO were characterized using multi-dimensional nuclear magnetic resonance (NMR) spectra and liquid chromatography-mass spectrometry (LC-MS). Shark cartilage KS was highly sulfated and modified with ~2.69% *N*-acetylneuraminic acid (NeuAc) through α(2,3)-linked to galactose. Additionally, KS exhibited binding affinity to *Ricinus communis* agglutinin I (RCA_120_) in a concentration-dependent manner, a highly toxic lectin from beans of the castor plant. Furthermore, KSO from dp2 to dp8 bound to RCA_120_ in the increasing trend while the binding affinity of dp8 was superior to polysaccharide. These results define novel structural features for KS from *Prionace glauca* cartilage and demonstrate the potential application on ricin-antidote exploitation.

## 1. Introduction

Glycosaminoglycans (GAGs) are anionic, linear polysaccharides consisting of repeating disaccharide units of hexuronic acid (d-glucuronic acid, GlcA, and/or its C5-epimer l-iduronic acid, IdoA) or hexose (d-galactose) and hexosamine (d-glucosamine, GlcN, or d-galactosamine, GalN). GAGs are categorized into four classes on the basis of variations in monosaccharide compositions, linkage types, and the degree and pattern of sulfated substitution: chondroitin sulfate (CS)/dermatan sulfate (DS), heparin (HP)/heparan sulfate (HS), hyaluronan (HA), and keratan sulfate (KS) [1,2].

KS is the only type of GAGs without uronic acid, and mainly composed of alternating repeating disaccharide unit of β (1,3)-linked galactose (Gal) and β (1,4)-linked *N*-acetylglucosamine (GlcNAc) with sulfation occurring at the C6 of both saccharide units [3,4,5]. In general, the degree of sulfation of KS varies in different tissues, such as cornea [6], cartilage [7], and brain [8]. In addition, minor but significant structural components, such as sialylation and fucosylation, are important modifications found in KS and essential for controlling the degradation of these molecules [9,10,11,12]. The *N*-acetylneuraminic acid (NeuAc) residues can be either α (2,3)- or α (2,6)-linked to Gal and typically occupy non-reducing terminal positions, while the fucose residues are α (1,3)-linked to GlcNAc [13].

Additionally, KS participates in diversely important biological processes in vivo. The high abundance of KS in cornea and cartilage is crucial in maintaining the proper hydration levels and keeps the cornea transparent [3,14]. In addition, KS in bones serves as a structural component endowed with cell binding properties [15]. KS proteoglycan with specific sulfation patterns, 6-*O*-sulfated-GlcNAc and non-sulfated Gal, is required in a critical period of visual cortex plasticity [16]. KS in microglia inhibits neural cell adhesion and the growth of spinal cord neurite in experimental autoimmune neuritis [17,18]. KS can also act as a diagnostic marker for carcinomas of the female genital tract [19]. Furthermore, KS oligosaccharides (KSO) also perform specific activities. KS disaccharide, Gal6Sβ1→4GlcNAc6S, inhibits interleukin 12 production by macrophages in murine Thy-1 type autoimmune disease [20]. Shirato et al. suggested KS disaccharide for treatment of airway inflammatory responses arising from bacterial infections [21]. KS disaccharide also prevents the progression of emphysema in murine models and is effective for treating chronic obstructive pulmonary disease [22].

Ricin is a potent cytotoxic glycoprotein derived from the beans of castor plant (*Ricinus communis*). It is composed of chain A and chain B, linked by a disulfide bond. Ricin A chain (32 kDa) is a ribosome-inactivating enzyme, while ricin B chain (34 kDa) is a galactose/*N*-acetylgalactosamine binding lectin [23]. The leading cause of death is that ricin can dissolve red blood cells and further cause the paralysis of cardiovascular and respiratory centers. Due to high toxicity and accessibility, ricin has been widely used as a terrorist weapon and for political assassination. It was reported that over 700 people have died of ricin poisoning. However, there is no antidote currently available for ricin poisoning. Thus, it is critical for the exploitation of antidote. *Ricinus communis* agglutinin I (RCA_120_) recognizes carbohydrate chains with non-reducing terminal β-d-galactose and Galβ1→4GlcNAc sequence [24]. Possessing the repeating structure of Galβ1→4GlcNAc, KS may be the candidate compound as an antidote.

Based on these various physiological and pharmacological activities, the structural characterization of KS is extremely important for developing structure–activity relationships and diagnosing related diseases. Two hydrolytic enzymes, keratanase I and keratanase II, are useful in facilitating the structural determination of KS. Additionally, nuclear magnetic resonance (NMR) and liquid chromatography-mass spectrometry (LC-MS) are all powerful techniques for structural elucidation. Multi-dimensional NMR spectra have been applied to characterizing the capping segments, main chain repeating unit and linkage region of KS derived from bovine tracheal cartilage [25]. Due to the soft ionization mode, electrospray ionization tandem mass spectrometry (ESI-MS/MS) has been useful for sequencing KSO obtained from various biological samples [26,27].

In the present study, a highly sulfated KS was isolated from *Prionace glauca* cartilage. The structure of KS and KSO generated through enzymatic hydrolysis were elucidated by NMR and hydrophilic interaction liquid chromatography-Fourier transform mass spectrometry (HILIC-FTMS) analysis. The interaction of KS and KSO with RCA_120_ was also determined using SPR and MOE docking methods, providing a foundation for further pharmaceutical exploitation of *Prionace glauca* cartilage KS.

## 2. Results and Discussion

### 2.1. Isolation and Chemical Composition Analysis

The crude polysaccharide from *Prionace glauca* cartilage was generated through proteolysis as previously reported [28]. The polysaccharides were separated on QFF column in gradient elution (Figure S1A); and two charged uniform fractions were acquired. Based on the enzymatic hydrolysis characteristics of the two fractions towards GAGs enzyme, Peak I was inferred to be CS and comprised of a mixture of CSA, CSC, and CSD sequences, as determined through disaccharide composition analysis (Figure S2, Table S2). Peak II was degraded by neither chondroitinase nor heparinase. However, according to the results of monosaccharide composition and its low uronic acid content, we concluded Peak II to be KS (Table S1). Single and symmetric peaks on both RID and MALLS indicated that CS and KS were of high purity (Figure S1B,C). The molecular weight of KS from shark cartilage (*M*_w_: 45,980) was much bigger than that from bovine corneal and chicken egg white (*M*_w_: 14,300 and 36,800, respectively), which might be related to the unique marine environment, such as low temperature; high pressure and salt; and lack of oxygen, light, and nutrition [29,30].

### 2.2. NMR Spectroscopy

The structural features of KS were properly characterized through a combination of 1D ^1^H-NMR, DEPTQ NMR, and 2D ^1^H-^1^H COSY, ^1^H-^13^C HSQC, as well as ^1^H-^13^C HMBC. Major ^1^H- and ^13^C-chemical shifts identified from these spectra were assigned as Table 1. The NMR profiles of KS sample were roughly consistent with the reported structure of KS [31].

**Figure 1:**
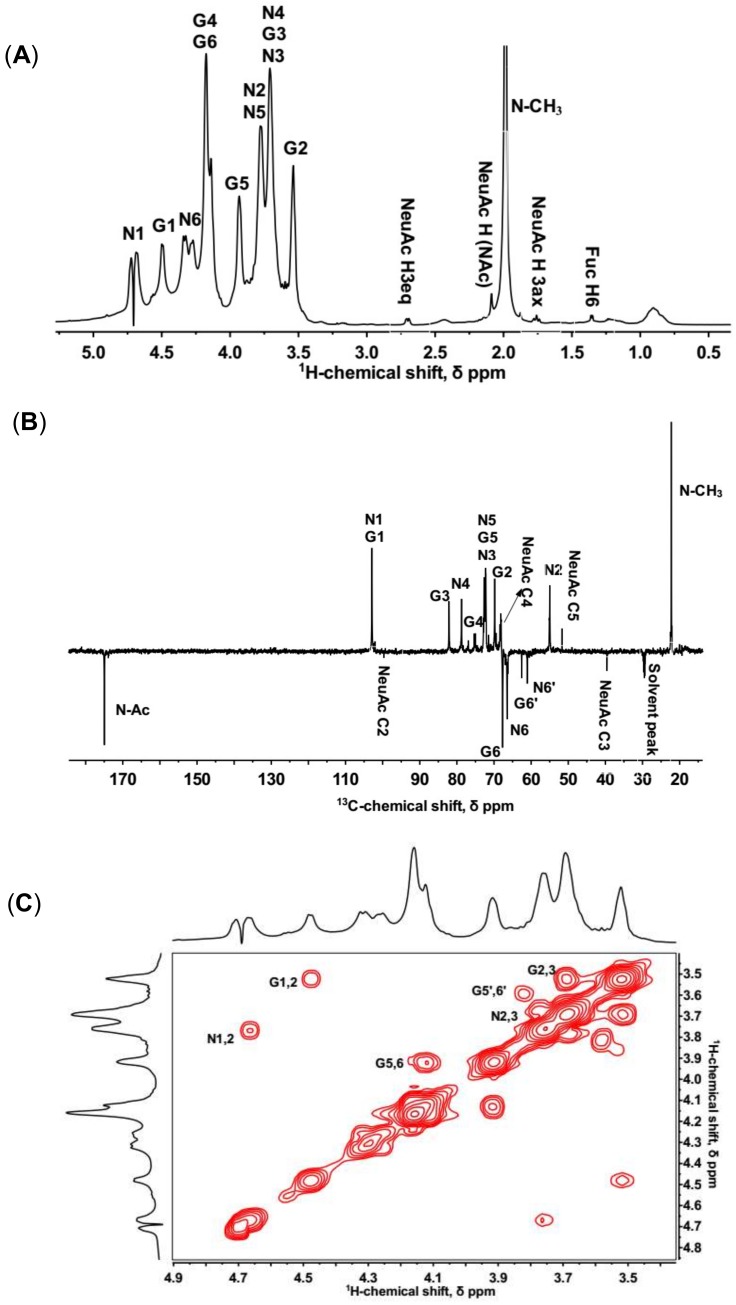

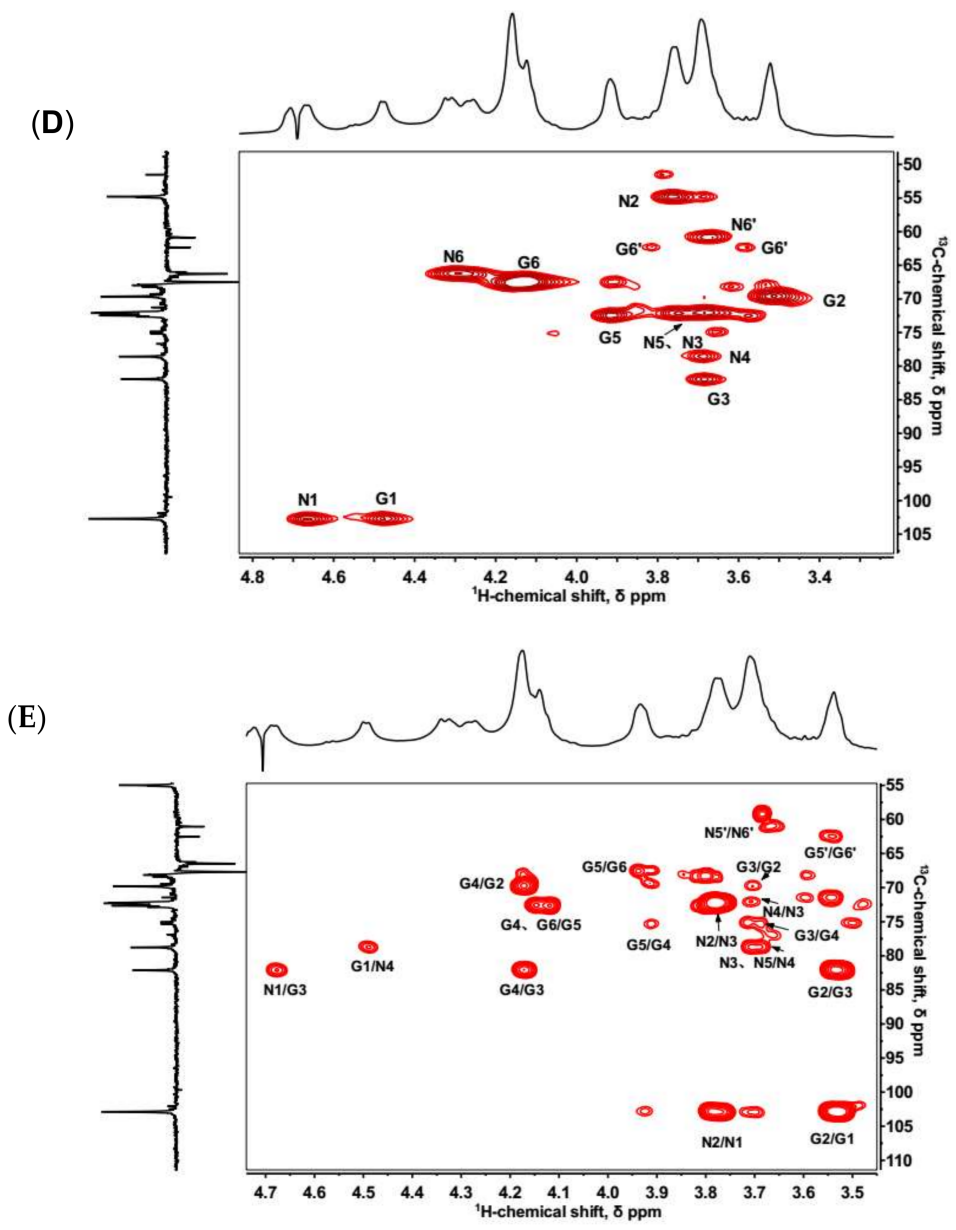
1D and 2D NMR spectra of isolated KS. N and G stand for GlcNAc and Gal, respectively. (**A**) Full ^1^H-NMR spectrum; (**B**) DPETQ spectrum; (**C**) ^1^H-^1^H COSY spectrum. G1,2 refers to a cross-peak between G H-1 and G H-2, etc. G5′,6′ refers to a cross-peak between non-sulfated G H-5 and G H-6. (**D**) ^1^H-^13^C HSQC spectrum. G1 refers to a cross-peak between G H-1 and G C-1, etc. G6′ refers to a cross-peak between non-sulfated G H-6 and G C-6, etc. (**E**) ^1^H-^13^C HMBC spectrum. G2/G1 refers to a cross-peak between G H-2 and G C-1, etc.

In ^1^H-NMR spectrum, KS had a crowded region between 3.4 and 4.8 ppm, resulting in a severe signal overlap for the majority of the resonances. Two notable anomeric proton signals at 4.66 and 4.48 ppm were identical with H-1 of GlcNAc and Gal, respectively (Figure 1A). The anomeric carbons at 102.68 and 102.73 ppm were deduced by DEPTQ NMR and ^1^H-^13^C HSQC (Figure 1B,D).

The non-reducing ends of KS chains are generally capped with NeuAc at the C-3 or C-6 of the terminal Gal [4]. In the ^1^H-NMR spectrum, signals at 1.74 and 2.68 ppm were consistent with H3ax and H3eq of NeuAc (Figure 1A). The signal at 39.38 ppm in ^13^C-NMR spectrum belonged to the unique C3 of NeuAc (Figure 1B). In addition, signals in the ^1^H-^1^H COSY and ^1^H-^13^C HSQC also demonstrated the presence of NeuAc (Figure S3).

Moreover, a signal of 1.33 ppm in the ^1^H-NMR spectrum corresponded to the H-6 of α (1,3)-linked fucose (Figure 1A). The ^1^H-^1^H COSY showed a connection from H-6 to H-5 at 1.33/4.27 ppm. A cross-peak at 1.33/19.16 ppm in the ^1^H-^13^C HSQC was assigned to the methyl group of fucose (Figure S3). Most signals of fucose were not identifiable since the low content of fucose resulted in signals covering by other sugars [32].

In ^1^H-^13^C HSQC, two characteristic β-anomeric ^1^H/^13^C-signals at 4.66/102.73 and 4.48/102.68 ppm were attributed to GlcNAc (denoted N1) and Gal (denoted G1) residues, respectively (Figure 1D). These signals were in approximately equimolar proportions, consistent with the disaccharide-repeating unit of KS. In the ^1^H-^13^C HMBC, two clear signals involved in glycosidic bonds were at 4.48/78.54 and 4.66/81.95 ppm (denoted N1/G3 and G1/N4, Figure 1E), which demonstrated β (1,3)- and β (1,4)-linkage types between GlcNAc and Gal, respectively. The signals of glycosidic bonds were also confirmed by ^1^H-^13^C HSQC due to the downfield shift of ^13^C-signals (denoted N4 and G3, Figure 1D).

Furthermore, in the ^1^H-^13^C HSQC, there were two cross-peaks at 4.29/66.21 and 4.13/67.49 ppm (denoted N6 and G6), assigned to 6-*O*-sulfo GlcNAc and 6-*O*-sulfo Gal residues, respectively (Figure 1D). Compared to the non-sulfated residues (denoted N6′ and G6′), these signals were shifted ~0.6 ppm downfield on the ^1^H-scale and ~5 ppm downfield on the ^13^C-scale. The results indicated that 6-sulfation primarily occurred at both of the repeating residues and the non-sulfated residues accounted for a very minor portion.

### 2.3. HILIC-FTMS Analysis of KS

**Figure 2:**
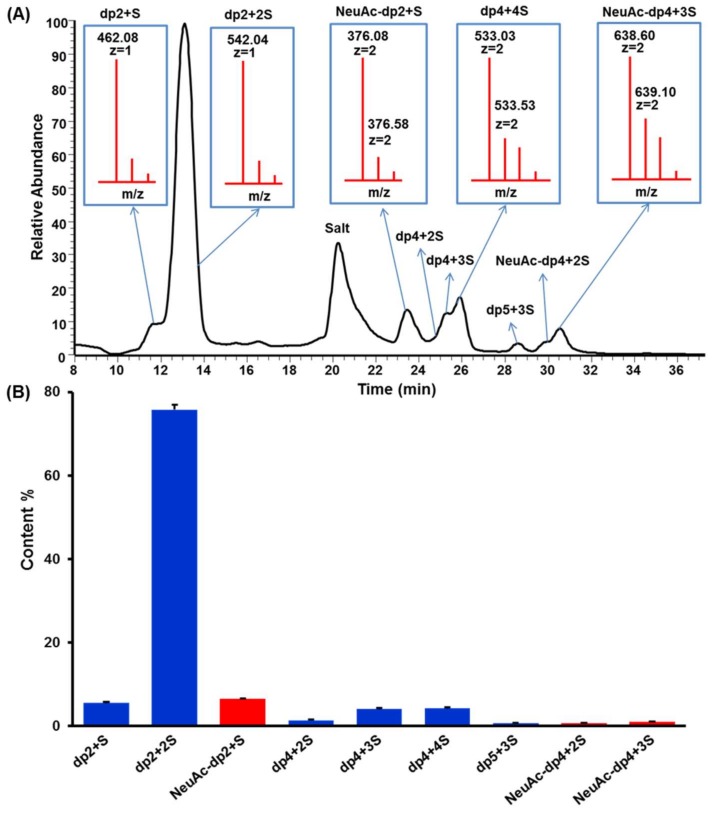
(**A**) Total ion chromatography of HILIC-FTMS profiling of fully digested KS domain structure. (**B**) Composition analysis of KSO calculated by GlycResoft. The analytical error for each GAG was <1%. The major composition unit of the oligomers was highly sulfated domains (approximately one sulfo group per saccharide). The minor component of NeuAc capped oligomers was also detected (degree of polymerization (dp)2 + sulfate (S), dp4 + 2S and dp4 + 3S with one NeuAc residue at the non-reducing end).

Keratanase II hydrolyzes β (1,3)-glucosaminidic linked to galactose in KS [33]. On cleavage, the enzyme requires a sulfo group at 6-*O*-position of GlcNAc but acts independently of a sulfo at 6-*O*-position of the Gal linked to the GlcNAc [34]. The KSO generated by keratanase II digestion were analyzed using HILIC-FTMS. The total ion chromatogram (TIC) of KSO sample was shown in Figure 2A. The results confirmed that the major repeating unit of the isolated KS was the disulfated disaccharide, (-3Gal6Sβ1→4GlcNAc6Sβ1-). The raw data was deconvoluted using DeconTools, and then the output of DeconTools was processed by GlycResoft to generate matching structures and to provide relative quantitative information [35]. A total of 31 oligosaccharides were matched by GlycResoft, which ranged from degree of polymerization (dp) 2 to dp 10 and included chains with both an even- and odd-number of sugar residues as well as sialylated oligosaccharides. Approximately 90% of the oligosaccharides were disaccharides. Relative quantitative results on the major oligosaccharides were shown graphically in Figure 2B. The extracted ion chromatograms of these major oligosaccharides were presented in Figure S4 and structural assignments relied on a mass accuracy of <5 ppm.

Moreover, the integration ratio between H3ax of NeuAc and H1 of Gal in ^1^H-NMR spectrum was 1:22, facilitating the calculation of NeuAc content as 2.69%, which closely agreed with the results calculated by GlycResoft. Based on the determined molecular weight and NeuAc content, we deduced that four NeuAc residues were present in each KS chain. This suggested that KS might have at least four branches since NeuAc was only believed to occupy the non-reducing ends of chains.

### 2.4. Preparation and Mapping of KSO

**Figure 3:**
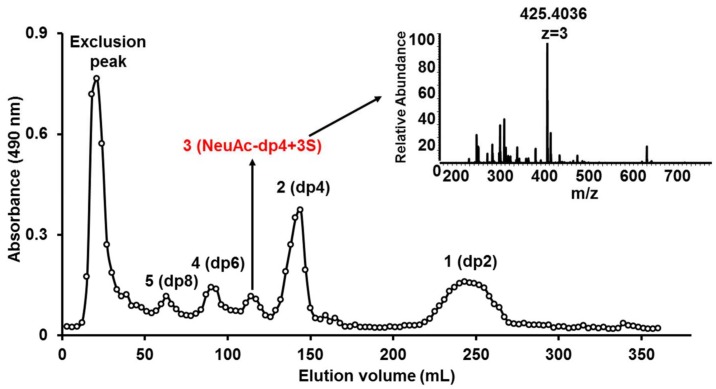
Elution profile of KSO on Bio-Gel P6 gel filtration chromatography.

KS sample was partly digested by keratanase II, and then fractionated on a Bio-Gel P6 column. The elution curve illuminated five prominent oligosaccharide fractions (Figure 3). Based on ESI-MS analysis, the five fractions corresponded to oligosaccharides of dp2, dp4, dp5, dp6, and dp8 (Figure S5). Surprisingly, oligosaccharide of dp5 was mainly a sialylated KS tetrasccharide with three sulfo-substitution.

**Figure 4:**
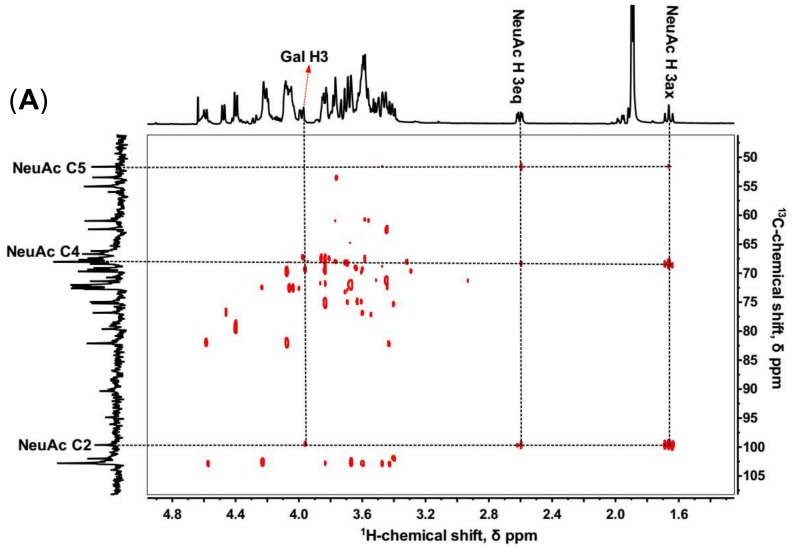

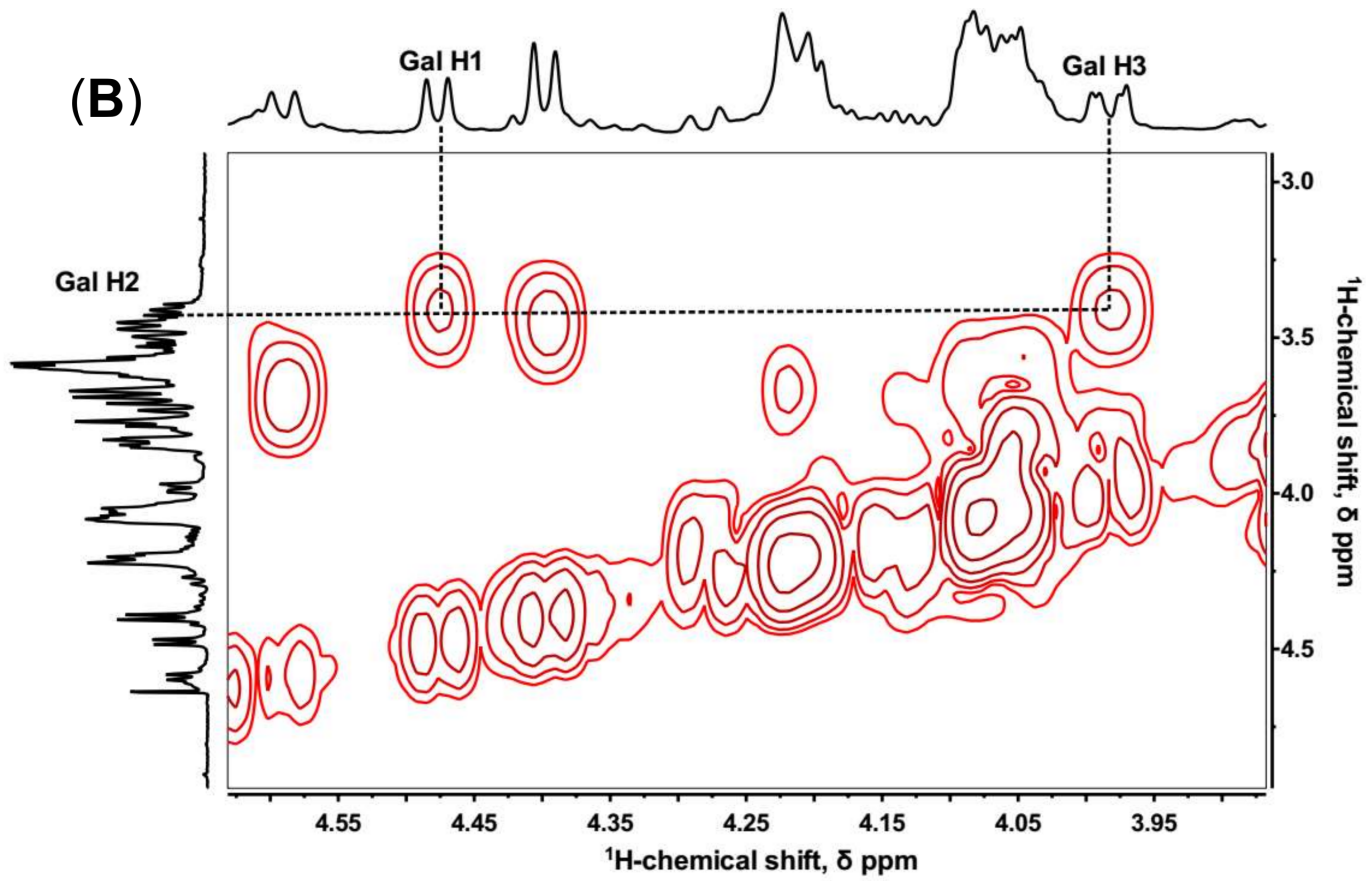
^1^H-^13^C HMBC (**A**) and ^1^H-^1^H COSY (**B**) of sialylated KS tetrasaccharide isolated from Bio-Gel P6 column.

The sialylated KS tetrasccharide was determined by 1D and 2D NMR spectroscopy (Figure 4) to confirm the linkage type between NeuAc and Gal. In ^1^H-^13^C HMBC, C2, C4, and C5 of NeuAc were assigned by the cross-peaks with H3ax and H3eq. Then the proton in Gal (H-X) correlating with the C2 of NeuAc was identified (Figure 4A), and the ^1^H-^1^H COSY showed H-X correlating with H-2 which also coupled with H-1 of Gal. Therefore, we inferred H-X was H-3, which demonstrated that NeuAc was α (2,3)-linked to Gal (Figure 4B). No sign of any α (2,6)-linkages was observed. However, in bovine articular cartilage, NeuAc α(2,3)- and α(2,6)-linked types were both identified [34].

**Figure 5:**
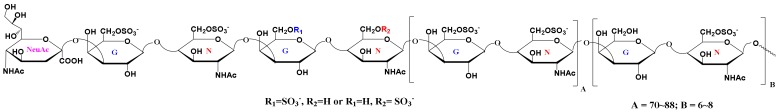
Schematic diagram of the chemical structure of KS.

Multistage mass spectrometric sequencing of sialylated KS tetrasccharide revealed that the unsulfated hexose might be Gal or GlcNAc in the reducing end (Figure S6). Thus, a generalized chemical structure of shark cartilage KS could be described (Figure 5).

These results demonstrated a higher degree of sulfation for *Prionace glauca* cartilage KS than KS derived from brain, bovine corneal, articular cartilage, and chicken egg white, all of which possessed less sulfation on the Gal residues [7,8,29,30]. Additionally, KS was modified with ~2.69% NeuAc through α(2,3)-linked to Gal.

### 2.5. RCA120 Binding Activities of KS and KSO

**Figure 6:**
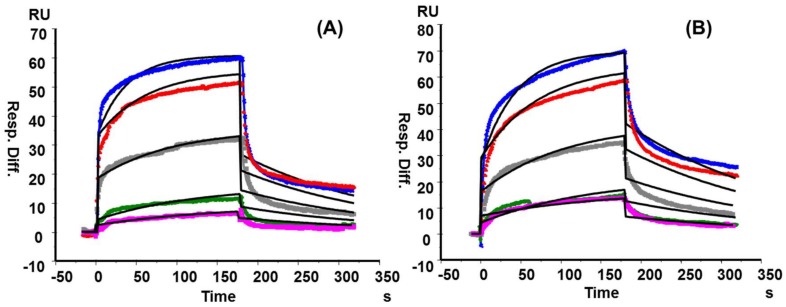
SPR sensor grams for interactions of RCA_120_ with KS from shark cartilage (**A**) and chicken egg white (**B**). The concentrations of each protein (from top to bottom): 500, 250, 125, 63, and 31 nM, respectively. The black curves were the fitting curves using models from BIAevaluate 4.0.1.

Although the strong binding properties among RCA_120_ and Galβ1→4GlcNAc (LacNAc) had been described previously through SPR, the interaction between RCA_120_ and KS polysaccharide in vitro was scarce [36,37,38]. However, our SPR results showed that both KS from shark cartilage and chicken egg white strongly bound to RCA_120_ in a concentration-dependent manner with *K_D_* values of 1.22 × 10^−7^ M and 1.37 × 10^−7^ M, respectively (Figure 6 and Table S3). Wang et al. once reported that RCA_120_ binding to Galβ1→4 linked oligosaccharide was enhanced by 2-*O*- or 6-*O*-sulfation but abolished by 4-*O*-sulfation [39]. However, concerning heterogeneity and flexibility of polysaccharide, the degree of sulfation at Gal residues hardly influenced RCA_120_ binding ability to a certain extent.

**Figure 7:**
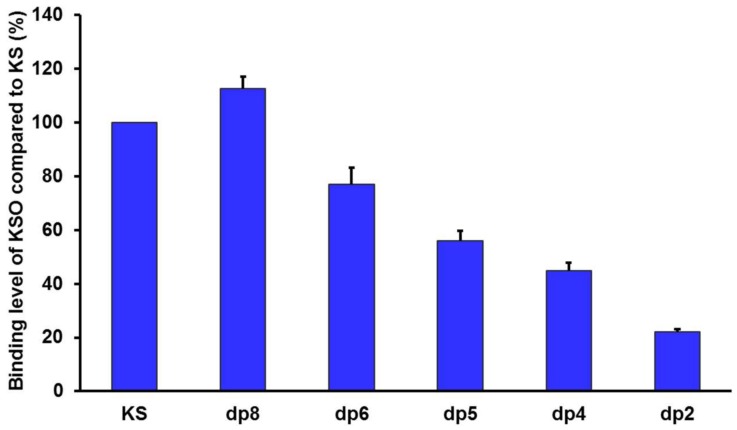
Normalized binding of shark cartilage KSO (from dp2 to dp8) and polysaccharide to RCA_120_. The concentrations of KSO (from dp2 to dp8) and polysaccharide were 1 mg/mL.

**Figure 8:**
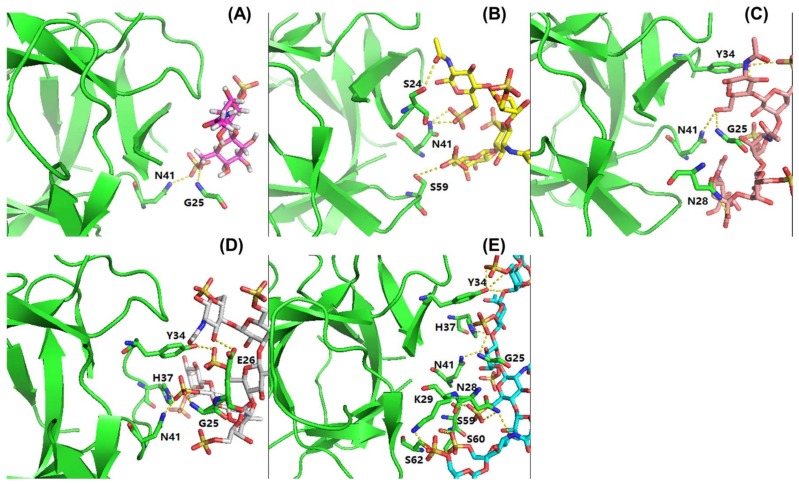
Binding modes of KSO to RCA_120_. (**A**–**E**) showed the binding modes of dp2, dp4, dp5, dp6, and dp8 to RCA_120_, respectively. The receptor was shown in green. The dashed lines showed hydrogen bonds between the ligands and the receptor.

In order to assess the influence of sugar chain length on RCA_120_ binding, KSO from dp2 to dp8 were eluted directly onto an SPR imaging chip at 1 mg/mL. Compared to KS, the relative binding strength was listed in Figure 7. As the results showed, along with the degree of polymerization increasing, the interaction with RCA_120_ was gradually enhanced. The conclusion was consistent with the MOE docking results in Table 2. The binding modes between RCA_120_ and KSO were shown as Figure 8. The growing numbers of hydrogen bond between the ligands and the receptor were accompanied by the extension of chain. Unexpectedly, the binding affinity of dp8 was superior to polysaccharide, which meant the potential application of oligosaccharide of dp8 in antidote exploitation.

## 3. Materials and Methods

### 3.1. Materials and Chemicals

*Prionace glauca* cartilage was obtained from Rushan Wantongming Biotech Company (Weihai, Shandong Province, China). The chondroitin ABC lyase and heparin lyases I, II, III were performed in our laboratory following the reported method [40]. XB-SAX chromatography column was from Welch, Shanghai. High performance gel permeation chromatography column (Shodex OHpak SB-804 HQ and SB-802.5 HQ) was from Showa Denko K.K., Tokyo, Japan. Packing materials for Q-Sepharose fast flow anion-exchange (QFF) column were from GE Healthcare Biosciences AB, Boston, MA, USA. Disaccharide standards of chondroitin sulfate were from Iduron, Cheshire, UK. Luna HILIC chromatography column was from Phenomenex, Torrance, CA, USA. Recombinant keratanase II expressed in *Escherichia coli* was prepared as previously described [33]. Acetonitrile and ammonium acetate were of HPLC grade (Sigma Aldrich, St. Louis, MO, USA). All other chemicals were of analytical grade.

### 3.2. Isolation and Purification of KS

KS from *Prionace glauca* cartilage was prepared by methods reported previously with minor modification [28]. The crude polysaccharide, generated by proteolysis, was separated on a QFF column, and eluted with a linear gradient of 0.7–2 mol/L NaCl at a flow rate of 4 mL/min. The eluate was tested by the phenol-sulfuric acid method at 490 nm [41]. The fraction containing polysaccharide was collected, concentrated, and desalted. The isolated fractions were then digested using chondroitin ABC lyase and heparinases (I, II, and III) and the products were detected by thin layer chromatography.

### 3.3. Molecular Weight and Chemical Composition Analysis

Uronic acid content was determined by modified carbazole method [42]. Sulfate content was determined by BaCl_2_-Gelatin method [43]. Purity and relative molecular weight (*M*_w_) were determined by high performance gel filtration chromatography system and detected by refractive index detector (RID) and multi-angle laser scattering system (MALLS) (Wyatt Technology, Santa Barbara, USA) [44]. Monosaccharide composition was determined by a pre-column 1-phenyl-3-methyl-5-pyrazolone (PMP) derivatization–HPLC method [45].

### 3.4. Profiling of KSO Generated by Keratanase II Digestion Through HILIC-FTMS

The KS sample was completely degraded by enzymatic hydrolysis. KS (100 µg) was dissolved in 100 μL of digestion buffer containing 50 mM ammonium acetate (pH 7.0). Excess keratanase II (50 mU) was added to KS sample and incubated at 37 °C overnight with gentle agitation. Enzymatic digestion was terminated by heating in a 100 °C water bath, and then spun down at 12,000 rpm for 5 min; supernatant was used directly for HILIC-FTMS analysis.

HILIC-FTMS analysis was performed on an Agilent 1290 LC ultra-performance liquid chromatography (UPLC) system (Agilent Technologies, Wilmington, DE, USA) equipped with a LTQ ORBITRAP XL mass spectrometer (Thermo, Scientific, Waltham, MA, USA). The KSO were separated by a Luna HILIC column (150 × 2.00 mm, 3 μm, Phenomenex) at 25 °C. The mobile phase was a mixture of 5 mM NH_4_OAc/98% acetonitrile (solvent A) and 5 mM NH_4_OAc/H_2_O (solvent B) at a flow rate of 150 μL/min. The gradient was programmed as 92% A initially and then linearly changed to 60% A over 58 min. The analysis was performed in the negative ion mode using a capillary temperature of 275 °C. The spray voltage was 4.2 kV and Nitrogen dry gas flowed at 40 L/min. Data acquisition and analysis were performed using Xcalibur 2.0 software (Thermo, Scientific, Waltham, MA, USA) and GlycReSoft 1.0 software (Publicly archived, http://code.google.com/p/glycresoft/downloads/list).

### 3.5. Preparation and Sequence Analysis of KSO

KS sample (200 mg) was partly digested by keratanase II (5 IU) at 37 °C for 5 h with gentle agitation. The reaction was terminated in 100 °C water bath, and the precipitant was removed by centrifugation at 12,000 r/min. The supernatant was loaded on Bio-Gel P6 column connected to an ÄKTA-fast protein liquid chromatography (FPLC) system (General Electric Company, Boston, MA, USA). The column was then eluted by 0.2 mol/L NH_4_HCO_3_ at a flow rate of 0.2 mL/min. Next, each fraction was analyzed by ESI-MS at the negative-ion mode. For collision induced dissociation (CID)-MS^n^ scanning, helium was used as collision gas with collision energy of 20–25 eV [46].

### 3.6. NMR Spectroscopy Analysis

Samples of KS and KSO were dissolved in 500 μL 99.9% deuterium oxide (D_2_O) respectively and freeze-dried three times to replace all exchangeable protons with deuterium, then redissolved in 500 μL D_2_O. One-dimensional (1D) ^1^H-NMR, DEPTQ NMR, and two-dimensional (2D) ^1^H-^1^H COSY, ^1^H-^13^C HSQC, ^1^H-^13^C HMBC were performed at 298K on Bruker BioSpin GmbH 600 MHz (Billerica, MA, USA) with Topspin 2.1.6 software (Bruker, Billerica, MA, USA). Chemical shifts were displayed relative to internal deuterated acetone for ^1^H and ^13^C.

### 3.7. Surface Plasmon Resonance (SPR) Binding Kinetics of RCA120-KS/KSO Interactions

SPR measurements were performed using a Biacore 3000 SPR instrument (General Electric Company, Boston, MA, USA). For the polysaccharides binding, biotinylated shark cartilage KS and chicken egg white KS (From lab of Robert J. Linhardt) [30] sensor chip were prepared by reaction of sulfo-*N*-hydroxysuccinimide long-chain biotin (Pierce, Rockford, IL, USA) with the free amino groups and the residue with the reducing end in the polysaccharide chain following a published procedure [29]. Two-fold serial dilutions of RCA_120_ were injected over the sensor chip at a flow-rate of 30 µL/min for a period of 3 min followed by 3 min dissociation period. For the oligosaccharides binding, RCA_120_ was immobilized to CM5 chip using amine coupling based on the manufacturer’s protocol. The successful immobilization of RCA_120_ was confirmed by the observation of a ~2000 RU increase in the sensor chip. KSO from dp2 to dp8 and polysaccharide (1 mg/mL) in HBS-EP buffer (0.01 M HEPES, 0.15 M NaCl, 3 mM EDTA, and 0.005% surfactant P20, (pH 7.4)) were injected at a flow rate of 30 µL/min for 3 min. At the end of the sample injection, the same buffer was flowed over the sensor surface to facilitate dissociation. The sensor chip was regenerated by injecting with 30 µL of 2 M NaCl and 30 µL of running buffer to get a fully regenerated surface. SPR experiments were performed in triplicate at each concentration, confirming reproducibility. The binding sensor grams (RU versus time) were pooled, trimmed, double referenced, and experimentally fit to different kinetic models using BIAevaluation software v4.0.1 (General Electric Company, Boston, MA, USA).

### 3.8. MOE Binding Affinity Calculation

Molecular docking was performed using MOE with the AMBER12: EHT force field. Ricin B-like lectin crystal structures used for the ADT calculations above were utilized. The induced fit docking approach was applied with consideration of the side chain flexibility of KSO at the binding site. The ligand binding site was defined using the bound ligands in the crystal structures. Ten docking conformations of the ligands were produced, and the best scored conformation with minimum binding energy was selected for analysis.

## 4. Conclusions

Herein, we reported a purified KS from *Prionace glauca* cartilage. A series of KSO were also obtained by KS digestion with keratanase II. The structure of KS and KSO were confirmed by multi-dimensional NMR spectra and HILIC-FTMS. The results showed that shark cartilage KS was highly sulfated and the major disaccharide repeating unit was -3Gal6Sβ1→4GlcNAc6Sβ1-. Moreover, this KS was modified by NeuAc capped non-reducing ends of chains, and the NeuAc was α (2,3)-linked to galactose.

SPR showed that KS bound to RCA_120_ in a concentration-dependent manner with the *K_D_* value of 1.22 × 10^−7^ M. Furthermore, KS oligosaccharides from dp2 to dp8 bound to RCA_120_ in the increasing trend, while the binding affinity of dp8 was superior to polysaccharide. MOE docking assays also verified the results. In conclusion, these results define novel structural features for KS from *Prionace glauca* cartilage and demonstrate the potential application in ricin-antidote exploitation.

## Figures and Tables

**Table 1:** Major ^1^H and ^13^C chemical shifts (in ppm) data for shark cartilage KS.

Signal/ppm	Nucleus	1	2	3	4	5	Sulfated C6	Unsulfated C6	CH_3_	C=O
4-β-GlcNAc-1 (N)	^1^H	4.66	3.77	3.67	3.69	3.75	4.29	3.67	1.97	-
	^13^C	102.73	54.82	72.04	78.54	72.13	66.21	61.06	22.24	174.97
3-β-Gal-1 (G)	^1^H	4.48	3.52	3.69	4.16	3.92	4.13	3.58	-	-
	^13^C	102.68	69.61	81.95	75.25	72.47	67.49	62.54	-	-

**Table 2:** The score of KSO predicted using MOEDock.

Ligands	Dock Score *
dp2	−8.70
dp4	−10.03
dp5	−10.58
dp6	−11.95
dp8	−14.01

* The score shows the values of the predicted binding energies (kcal/mol) using MOEDock.

## Supplementary Materials

The following are available online at http://www.mdpi.com/1660-3397/16/4/128/s1, Table S1: Physicochemical properties analysis of KS and CS, Table S2: Disaccharides composition of CS, Table S3: Summary of kinetic data of shark KS and egg KS-RCA_120_ interactions, Figure S1: Elution profile of GAGs from *Prionace glauca* cartilage on a QFF ion-exchange column, Figure S2: Separation chromatography of CS disaccharides analysis on SAX-HPLC, Figure S3: NMR signals of NeuAc and fucose in KS, Figure S4: The extracted ion chromatograms (EICs) of KSO based on HILIC–MS analysis, Figure S5: Negative-ion mass spectra of KS oligosaccharides isolated by Bio-Gel P6, Figure S6: Negative-ion ESI-MS^n^ product-ion spectra of sialylated KS tetrasaccharide (Sia-dp4+3S) isolated by Bio-Gel P6.
