# Supplementary material for: Structural Characterization and Interaction with RCA120 of a Highly Sulfated Keratan Sulfate from Blue Shark (Prionace glauca) Cartilage
Source: Mar Drugs. 2018 Apr 14;16(4):128. doi: 10.3390/md16040128 (PMC5923415; doi:10.3390/md16040128)
Supplement: Supplementary file 1 [file marinedrugs-16-00128-s001.pdf]

marine drugs

Supplementary Materials: Structural Characterization and Interaction with RCA120 of a Highly Sulfated Keratan Sulfate from Blue Shark (*Prionace glauca*) Cartilage

Table S1 Physicochemical properties analysis of KS and CS.

Sample	Yield (%)	Uronic acid (%)	Sulfate (%)	Protein (%)	Molecular weight (kDa)			Monosaccharide composition (%)			
					Mw	*Mn*	*Mw/Mn*	GlcN	GlcA	Gal	GalN
KS	21.8	3.5	26.1	6.0	45.98	34.62	1.33	49.4	—	50.6	—
CS	46.9	36.8	24.0	--	38.49	32.49	1.18	—	40.2	—	59.8

Table S2 Disaccharides composition of CS.

CS/DS disaccharides	0S	6S	4S	2,6S	2,4S
Content%	1.08	39.54	25.21	32.60	1.58

Table S3 Summary of kinetic data of shark KS and egg KS-RCA120 interactions.

Interactions	k_a (1/MS)	k_d (1/S)	K_D (M)
Shark KS	4.42×10^{4}	5.41×10^{-3}	1.22×10^{-7}
Egg KS	3.63×10^{4}	4.98×10^{-3}	1.37×10^{-7}

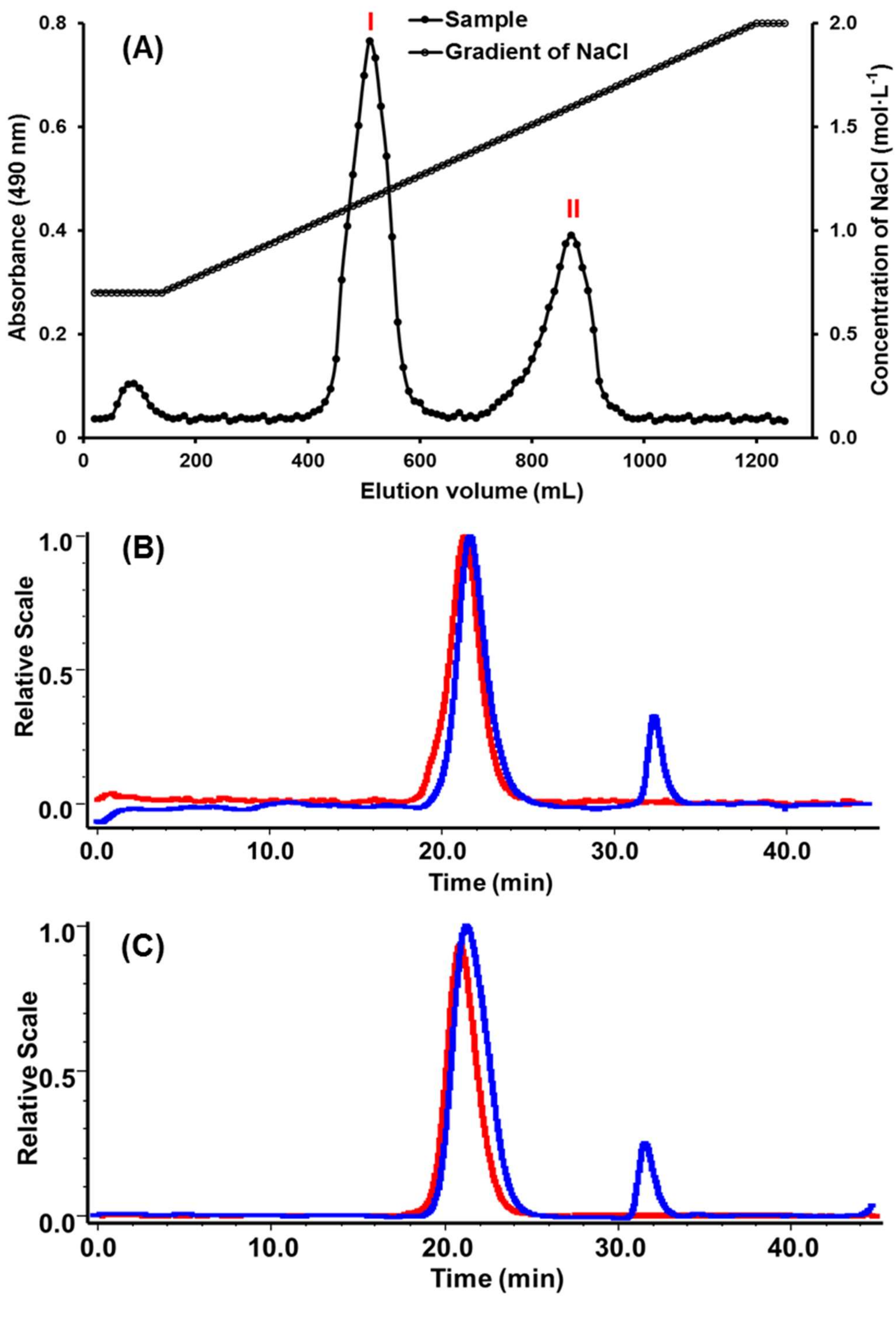

Figure S1. Elution profile of GAGs from *Prionace glauca* cartilage on a QFF ion-exchange column (A). Peak I was eluted with 1.2 M NaCl solution, while Peak II was eluted with 1.6 M NaCl solution. Molecular weight determination of CS (B) and KS (C). The x-axes correspond to elution time and the y-axes correspond to the signals detected using RID (Blue line) and MALLS (Red line).

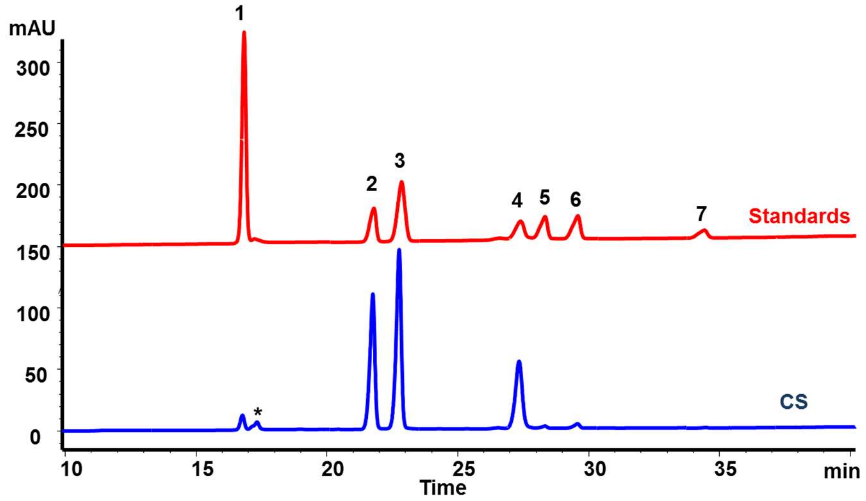

Figure S2. Separation chromatography of CS disaccharides analysis on SAX-HPLC. The red line represents seven disaccharide standards. Numbered peaks correspond to known disaccharide standards as follows: 1, ΔDi-0S; 2, ΔDi-6S; 3, ΔDi-4S; 4, ΔDi-2,6S; 5, ΔDi-4,6S; 6, ΔDi-2,4S; 7, ΔDi-2,4,6S; Peaks labeled with "*" were contaminants from the reaction system.

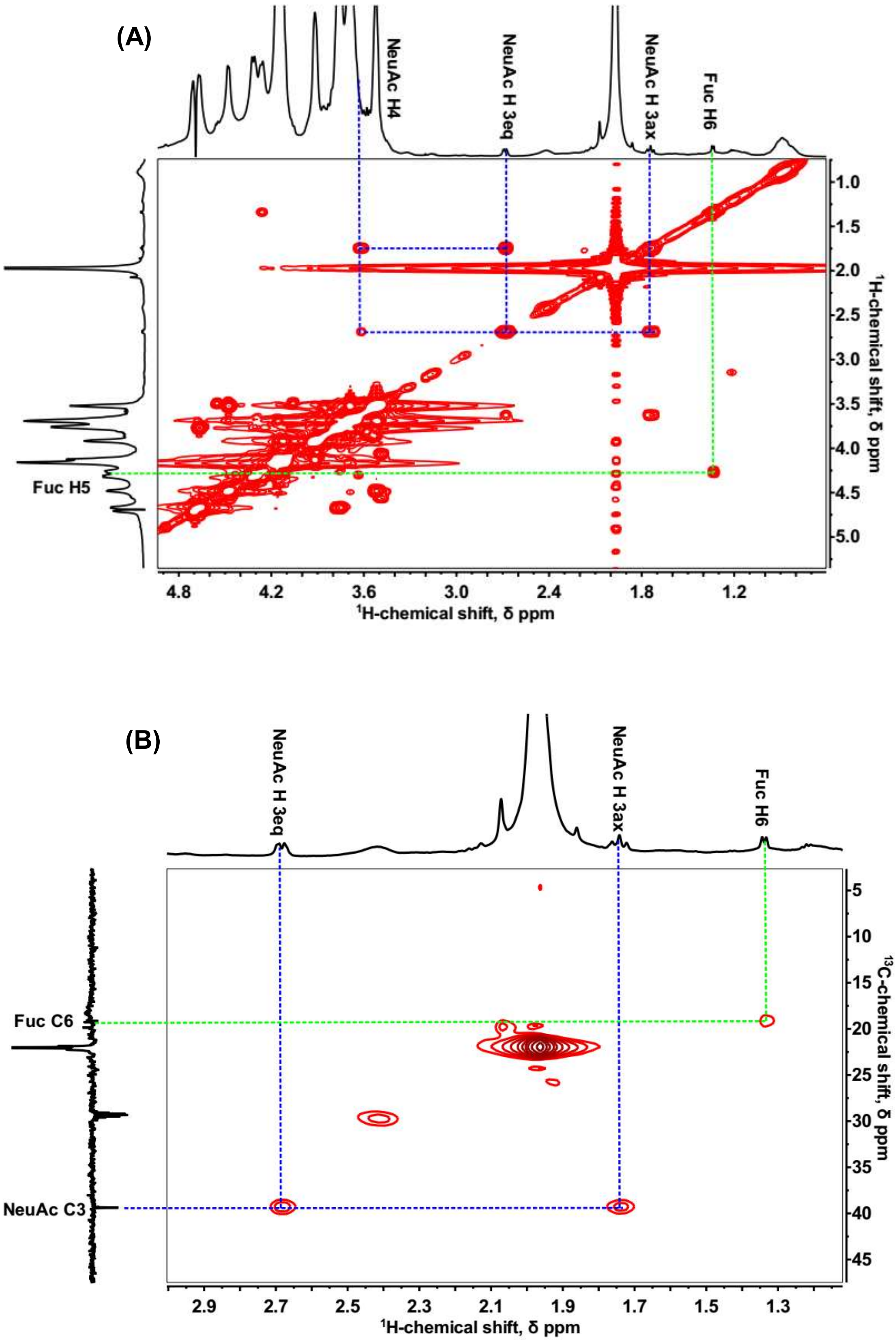
(A)
NeuAc H4
NeuAc H 3eq
NeuAc H 3ax
Fuc H6
Fuc H5
1H-chemical shift, δ ppm
4.8
4.2
3.6
3.0
2.4
1.8
1.2
1.0
1.5
2.0
2.5
3.0
3.5
4.0
4.5
5.0
(B)
NeuAc H 3eq
NeuAc H 3ax
Fuc H6
Fuc C6
NeuAc C3
13C-chemical shift, δ ppm
1H-chemical shift, δ ppm
2.9
2.7
2.5
2.3
2.1
1.9
1.7
1.5
1.3
5
10
15
20
25
30
35
40
45

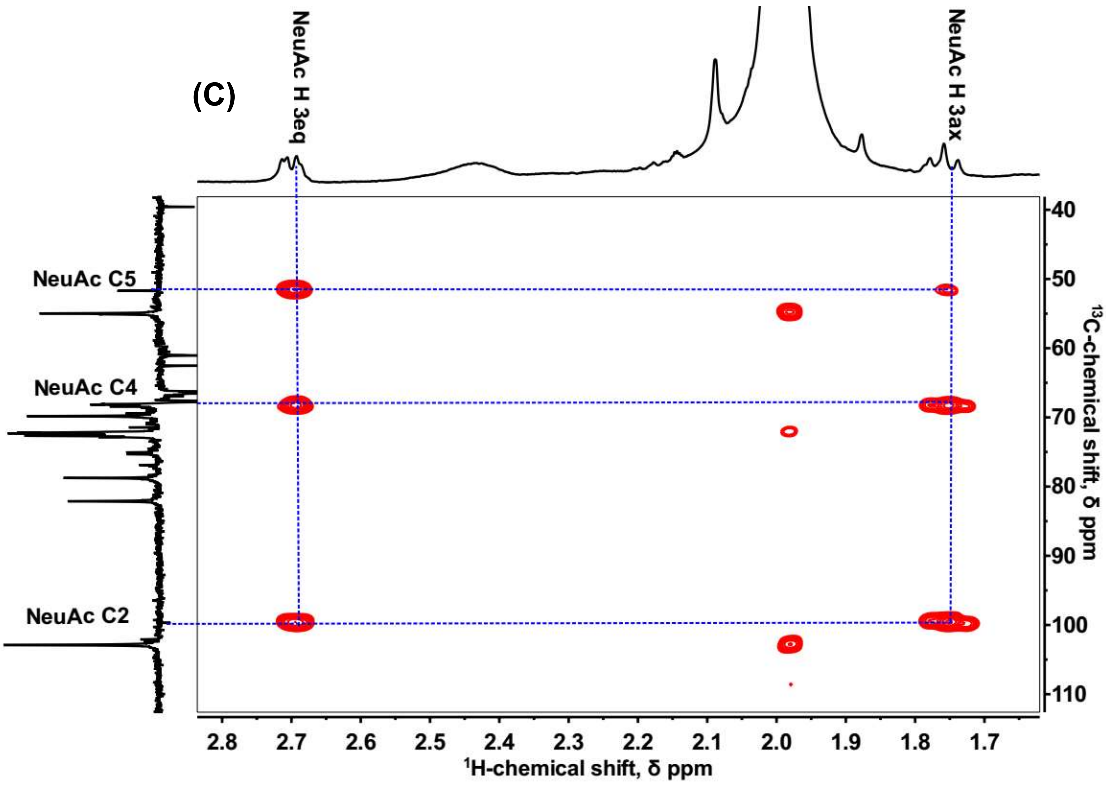

Figure S3. Signals of NeuAc and fucose in KS determined byp 2D ^{1}H-^{1}H- COSY (A), ^{1}H-^{13}C HSQC (B) and ^{1}H-^{13}C- HMBC (C).

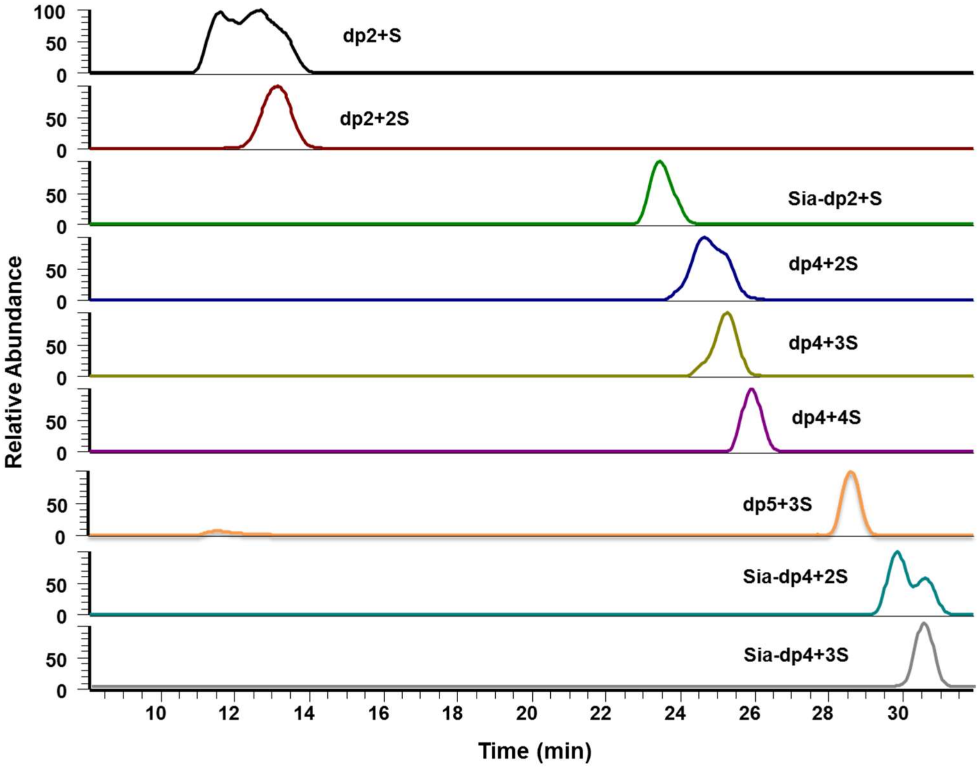

Figure S4. The extracted ion chromatograms (EICs) of KSO based on HILIC–MS analysis.

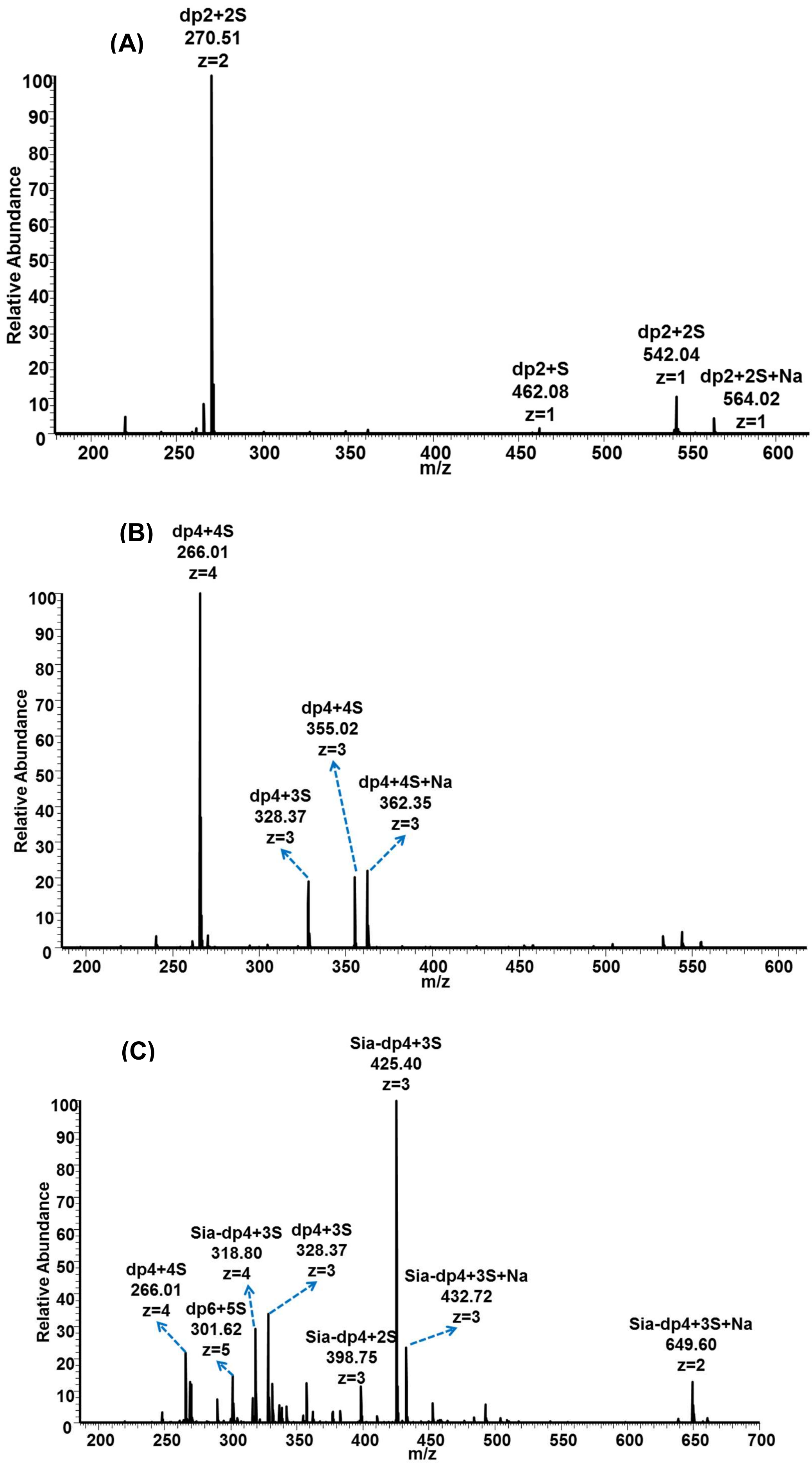
(A)
dp2+2S
270.51
z=2
dp2+S
462.08
z=1
dp2+2S
542.04
z=1
dp2+2S+Na
564.02
z=1
Relative Abundance
m/z
(B)
dp4+4S
266.01
z=4
dp4+3S
328.37
z=3
dp4+4S
355.02
z=3
dp4+4S+Na
362.35
z=3
Relative Abundance
m/z
(C)
Sia-dp4+3S
425.40
z=3
dp4+4S
266.01
z=4
dp6+5S
301.62
z=5
Sia-dp4+3S
318.80
z=4
dp4+3S
328.37
z=3
Sia-dp4+2S
398.75
z=3
Sia-dp4+3S+Na
432.72
z=3
Sia-dp4+3S+Na
649.60
z=2
Relative Abundance
m/z

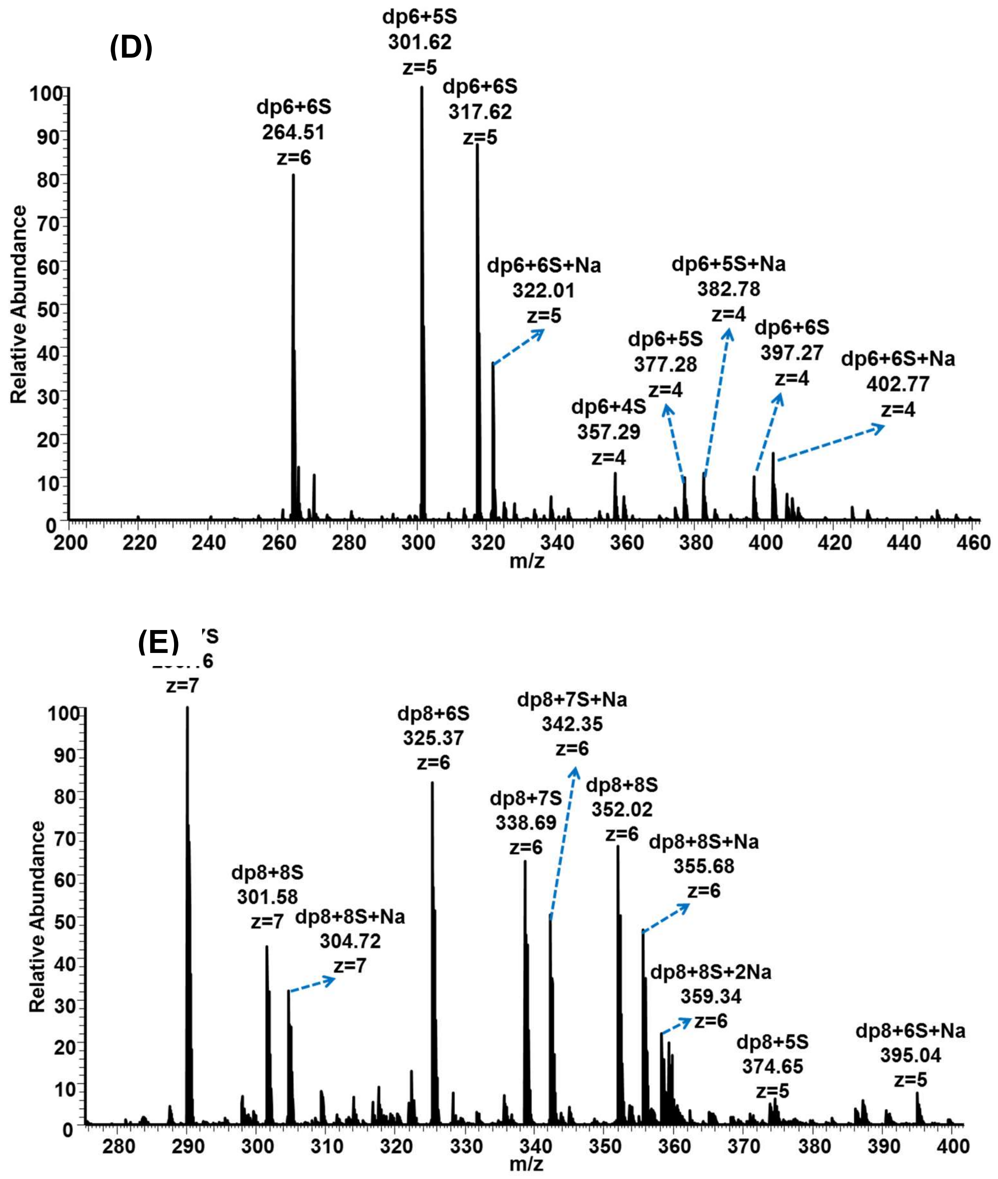

Figure S5. Negative-ion mass spectra of KS oligosaccharides isolated by Bio-Gel P6. (A) Fraction 1, dp2; (B) Fraction 2, dp4; (C) Fraction 3, dp5; (D) Fraction 4, dp6; (E) Fraction 5, dp8. "dp" represents degree of polymerization; "S" represents sulfate.

MS[1.]

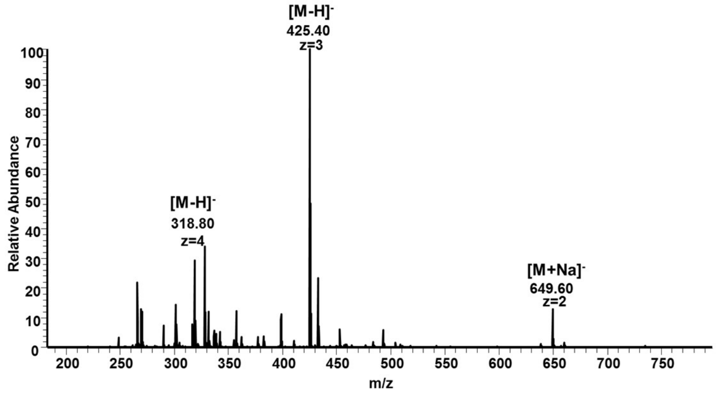

The first structure:

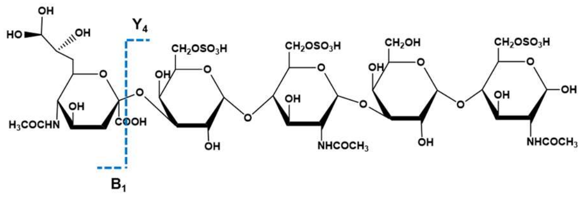

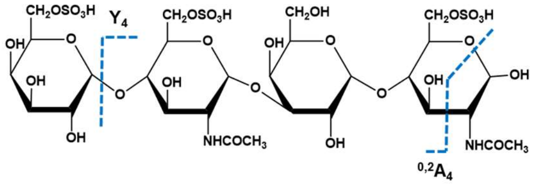

MS^2 of 425.40^{3-}→ MS^3 of 425.40^{3-}→328.37^{3-}→

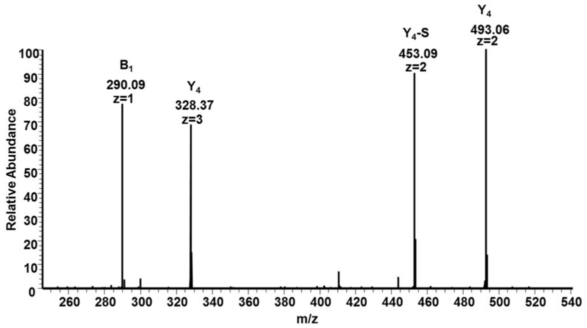

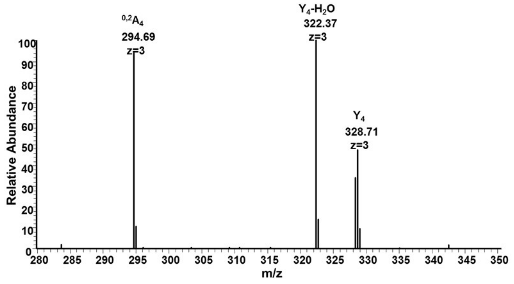

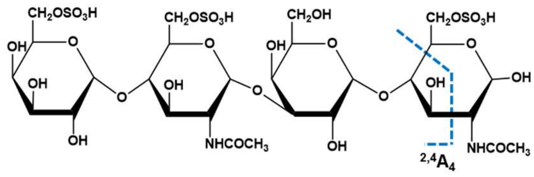

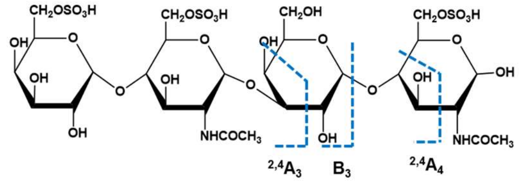

MS^4 of 425.40^{3-}→328.37^{3-}→294.69^{3-}→MS^5 of 425.40^{3-}→328.37^{3-}→294.69^{3-}→372.55^{2-}→

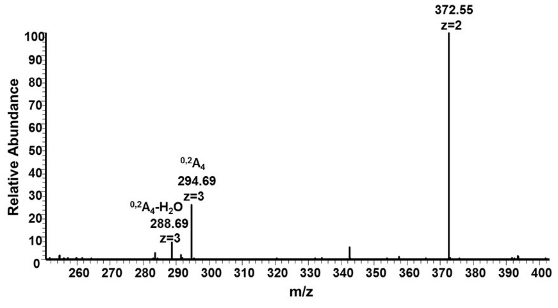

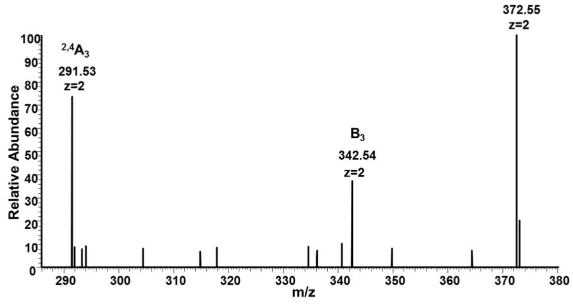

The second structure:

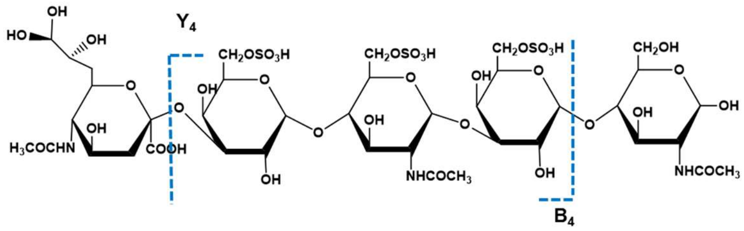

MS[2] of 318.80^{3-}→

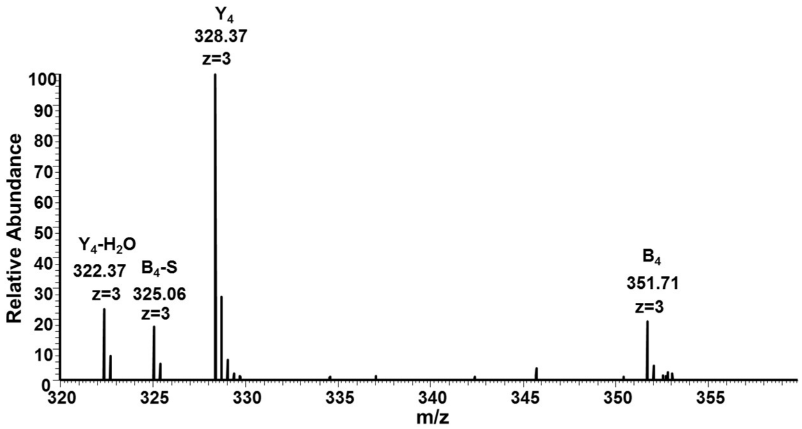

Figure S6. Negative-ion ESI-MS[n] product-ion spectra of sialylated KS tetrasaccharide (Sia-dp4+3S) isolated by Bio-Gel P6. "dp" represents degree of polymerization. "S" represents sulfate.
